# Correction: Association between cancer stigma and cervical cancer screening uptake among women of Dhulikhel and Banepa, Nepal

**DOI:** 10.1371/journal.pone.0318367

**Published:** 2025-01-24

**Authors:** Bandana Paneru, Aerona Karmacharya, Alina Bharati, Soniya Makaju, Bikram Adhikari, Dikshya Kafle, Sunila Shakya, Spiegelman Donna, Sangini Seth, Anne Stangl, Aamod Dhoj Shrestha, Archana Shrestha

The eight author’s full name is incorrect. The correct full name is: Spiegelman Donna.
